# Continuous Production of Ethanol, 1-Butanol and 1-Hexanol from CO with a Synthetic Co-Culture of *Clostridia* Applying a Cascade of Stirred-Tank Bioreactors

**DOI:** 10.3390/microorganisms11041003

**Published:** 2023-04-12

**Authors:** Miriam Bäumler, Veronika Burgmaier, Fabian Herrmann, Julian Mentges, Martina Schneider, Armin Ehrenreich, Wolfgang Liebl, Dirk Weuster-Botz

**Affiliations:** 1 Chair of Biochemical Engineering, Department of Energy and Process Engineering, TUM School of Engineering and Design, Technical University of Munich, 85748 Garching, Germany; miriam.baeumler@tum.de (M.B.); veronika.burgmaier@tum.de (V.B.); fabian.herrmann@tum.de (F.H.); julian.mentges@tum.de (J.M.); 2 Chair of Microbiology, TUM School of Life Sciences, Technical University of Munich, 85354 Freising, Germany; martina.schneider@tum.de (M.S.); aehrenr@tum.de (A.E.); wliebl@tum.de (W.L.)

**Keywords:** *C. kluyveri*, *C. carboxidivorans*, chain elongation, syngas fermentation, carbon monoxide, bioreactor cascade, continuous alcohol production

## Abstract

Syngas fermentation with clostridial co-cultures is promising for the conversion of CO to alcohols. A CO sensitivity study with *Clostridium kluyveri* monocultures in batch operated stirred-tank bioreactors revealed total growth inhibition of *C. kluyveri* already at 100 mbar CO, but stable biomass concentrations and ongoing chain elongation at 800 mbar CO. On/off-gassing with CO indicated a reversible inhibition of *C. kluyveri*. A continuous supply of sulfide led to increased autotrophic growth and ethanol formation by *Clostridium carboxidivorans* even at unfavorable low CO concentrations. Based on these results, a continuously operated cascade of two stirred-tank reactors was established with a synthetic co-culture of both *Clostridia.* An amount of 100 mbar CO and additional sulfide supply enabled growth and chain elongation in the first bioreactor, whereas 800 mbar CO resulted in an efficient reduction of organic acids and de-novo synthesis of C2-C6 alcohols in the second reactor. High alcohol/acid ratios of 4.5–9.1 (*w*/*w*) were achieved in the steady state of the cascade process, and the space-time yields of the alcohols produced were improved by factors of 1.9–5.3 compared to a batch process. Further improvement of continuous production of medium chain alcohols from CO may be possible by applying less CO-sensitive chain-elongating bacteria in co-cultures.

## 1. Introduction

Synthesis gas (syngas) is a by- or waste product of many industrial processes, such as steel-mill off-gases, or it can be produced from organic residues by gasification of carbon rich materials. Anaerobic acetogenic microorganisms can convert this syngas with the main compounds CO, CO_2_ and H_2_ to alcohols like ethanol or other platform chemicals by syngas fermentation processes [1,2,3,4,5]. The added value of the fermentation product increases with the number of carbon atoms. For example, hexanoate/hexanol has twice the market value of ethanol [6]. In addition to the higher added value, the extraction of medium-chain fatty acids is less costly due to their lower water solubility in contrast to short-chain fatty acids or ethanol [7,8]. However, the production rates and concentrations of butyrate/1-butanol or hexanoate/1-hexanol in gas fermentations with acetogenic microorganisms are low [9]. Microbial formation of medium-chain carboxylic acids from acetate and ethanol, the main products of syngas fermentations with acetogenic microorganisms, can be achieved by chain elongation using other anaerobic bacteria and has attracted increasing interest in recent years [10,11,12,13]. *Clostridium kluyveri* is the best-studied chain-elongating bacterium that can convert a 2:1 mixture of ethanol and acetate to butyrate, hexanoate, hydrogen gas (H_2_) and even small amounts of octanoate [14,15].

Batch processes with a defined co-culture of *Clostridium autoethanogenum* and *C. kluyveri* in anaerobic shaken bottles with an initial CO partial pressure of 1.1 bar CO in the headspace resulted in final product concentrations of 2.3 g L^−1^ butyrate, 0.7 g L^−1^ hexanoate, 0.7 g L^−1^ 1-butanol, and 0.4 g L^−1^ 1-hexanol after 12 days [16]. By doubling the process time, supplementing with acetate and omitting shaking for the first 4 days, the concentrations of the fatty acids were increased [16]. In stirred-tank bioreactor batch experiments at pH 7.5 with continuous gassing of 30% CO, 5% CO_2_, 15% H_2_ and 50% N_2_, a co-culture consisting of *Clostridium aceticum* and *C. kluyveri* resulted in maximum concentrations of 7.0 g L^−1^ butyrate, 8.2 g L^−1^ hexanoate, and 0.7 g L^−1^ 1-butanol after a process time of 900 h by using additional ethanol supply [17]. Final concentrations of 1.2 g L^−1^ butyrate/1-butanol and 0.6 g L^−1^ hexanoate/1-hexanol were measured in batch processes applying a continuously gassed stirred-tank bioreactor with 80% CO and 20% CO_2_ for the co-cultivation of *Clostridium carboxidivorans* and *C. kluyveri* at pH 6.0 after a process time of 144 h [18]. Two specific 23S rRNA-targeted oligonucleotide probes, which were designed and verified for both strains to enable specific labeling of fermenter samples by fluorescence in situ hybridization (FISH) [19], enabled the monitoring of both strains during co-cultivation. This measurement of the individual cell concentrations in the co-culture revealed a restricted growth of *C. kluyveri* due to a mismatching pH and the high initial CO partial pressure [18]. Nevertheless, the space-time yields revealed an increase in alcohol production rates in particular with the co-culture consisting of *C. carboxidivorans* and *C. kluyveri*: 1-butanol was produced at 0.14 g L^−1^ d^−1^ and 1-hexanol at 0.04 g L^−1^ d^−1^ compared to 0.06 g L^−1^ d^−1^ and 0.03 g L^−1^ d^−1^, respectively, reported by Diender et al., or 0.02 g L^−1^ d^−1^ 1-butanol without any 1-hexanol production as recently reported by Fernández-Blanco et al. [16,17].

Since continuously operated bioprocesses are an option to further increase the space-time yields, the chain elongation of a monoculture of *C. kluyveri* was investigated in a continuously operated stirred-tank bioreactor with the effluent of a syngas fermentation with *C. autoethanogenum* as influent. The continuous process with added growth factors such as trace elements resulted in carbon conversions of 90% with *C. kluyveri* at pH 7.0 and achieved a hexanoate production with a space-time yield of 4.1 g L ^−1^ d ^−1^ [11].

Based on these promising results of continuous processing, the continuous alcohol production from CO/CO_2_ will be studied in the present work by co-culturing of *C. carboxidivorans* and *C. kluyveri*. The acetogen *C. carboxidivorans* will be applied for the conversion of CO/CO_2_ to acetate and ethanol and for the reduction of the organic acids butyrate and hexanoate produced by the chain-elongating *C. kluyveri*. As the formation of acetate and ethanol by *C. carboxidivorans* is favored at pH 6.0 (acetogenesis), whereas the reduction of organic acids to the corresponding alcohols (solventogenesis) needs lower pH, as already shown with a mono-culture of *C. carboxidivorans* [20], a cascade of two stirred-tank reactors in a series with varying pH was considered to be beneficial.

Besides the selection of a suitable pH for the co-cultivation, the partial pressure of CO seems to be a critical process variable, as the metabolism of a pure culture of *C. kluyveri* is already inhibited at low CO partial pressures, as was shown in anaerobic shaking bottle experiments [16]. On the other hand, many acetogens require high CO partial pressures in the gas phase to avoid CO limitation and reduced alcohol production rates [2]. As a consequence, we will investigate the effects of varying CO partial pressures in batch operated stirred-tank bioreactors with continuous gassing at increased gas-liquid mass transfer rates with a mono-culture of *C. kluyveri*.

Recently it was shown that continuous sulfide supply enhanced the autotrophic production of alcohols with *Clostridium ragsdalei*, allowing ethanol concentrations to increase more than 3-fold to 7.67 g L^−1^ and the alcohol-to-acetate ratio to increase 43-fold to 17.71 g g^−1^ [21]. As *C. kluyveri* needs a 2:1 mixture of ethanol and acetate for the chain elongation to butyrate and hexanoate [14,15], a continuous sulfide supply will be studied, as a preliminary step, in batch-operated stirred-tank reactors with continuous gassing with a mono-culture of *C. carboxidivorans* to check for an improvement of the ethanol-to-acetate ratio.

Suitable reaction conditions (CO partial pressures and sulfide supply rates) were transferred afterwards to the cascade of two stirred-tank reactors in a series to reach the main purpose of this study: continuous alcohol production from CO/CO_2_ with a defined co-culture of *C. carboxidivorans* and *C. kluyveri*.

## 2. Materials and Methods

### 2.1. Microorganisms and Medium

*C. carboxidivorans* (DSM 15243) and *C. kluyveri* (DSM 555) were obtained from the German Collection of Microorganisms and Cell Cultures DSMZ (Braunschweig, Germany). The detailed medium composition is listed in the Supplementary Materials (Table S1). The medium was anaerobized through boiling and subsequent gassing with N_2_. Cysteine hydrochloride was added prior to inoculation from a previously anaerobized and sterilized stock solution.

### 2.2. Preculture Preparations

Each strain received from the DSMZ (Braunschweig, Germany) was first grown in a medium, then supplemented with glycerol and stored as frozen stock culture at −80 °C until they were needed for preculture preparation. For preculture preparation, 2.5 mL of a frozen stock culture was thawed and then inoculated in anaerobic flasks containing N_2_ in the headspace at a total pressure of 1.0 bar via a septum (butyl rubber stopper, Glasgerätebau Ochs, Bovenden, Germany) using a single-use syringe (BD Discardit II, Becton Dickinson, Franklin Lakes, NJ, USA) and sterile needles (Sterican 0.9 × 70 mm, B. Braun, Melsungen, Germany).

*C. carboxidivorans* was grown heterotrophically in 500 mL anaerobic flasks with 100 mL medium supplemented with 5 g L^−1^ glucose as a carbon source at 37 °C in a shaking incubator (Wisecube WIS-20R, witeg Labortechnik GmbH, Wertheim, Germany) at 100 rpm and an eccentricity of 2.5 cm for 21 h.

*C. kluyveri* was grown heterotrophically in 500 mL anaerobic flasks with 100 mL medium with the addition of 10 g L^−1^ potassium acetate and 20 mL L^−1^ ethanol as carbon sources at 37 °C and shaken at 100 rpm and an eccentricity of 2.5° m for 110 h. Concentrations of the carbon sources and the reducing agent were adopted as described for the DSM-52 medium (DSMZ, Braunschweig, Germany), whereby sodium sulfide was replaced with 0.5 g L^−1^ cysteine-HCl. 2.5 g L^−1^ Na_2_HCO_3_ were added for providing the HCO_3_/CO_2_ necessary for unimpeded growth of *C. kluyveri* [22,23].

Cells were harvested in the exponential growth phase by centrifugation (10 min, 3620 rcf, Rotica 50 RS, Hettich GmbH & Co., KG, Tuttlingen, Germany) and resuspended in anaerobic phosphate-buffered saline (PBS, pH 7.4) for inoculation of the stirred-tank bioreactor.

### 2.3. Stirred-Tank Bioreactor Set-Up for Gas-Fermentation Processes

All batch processes were carried out either in a fully controlled anaerobic 2-L stirred-tank reactor (STR) (Labfors, Infors AG, Bottmingen, Switzerland) with a working volume V_R_ of 1 L or in a fully controlled and in situ sterilisable, cylindrical 3.7-L stirred-tank bioreactor (KLF3000, Bioengineering AG, Wald, Switzerland) with a working volume V_R_ of 2 L. Both were agitated with two Rushton turbines at 1200 rpm (volumetric power input P V^−1^ of 11.7 W L^−1^ (working volume of 1 L), and 10.5 W L^−1^ (working volume of 2 L), respectively). Temperature was controlled at 37 °C, and pH was controlled by using either NaOH (3 M) or H_2_SO_4_ (1 M). The STRs were continuously gassed with gassing rates of 5 L h^−1^ (resulting in 0.083 vvm on a 1 L scale and 0.042 vvm on a 2 L scale) and individual gas mixtures of CO:CO_2_:N_2_ controlled by independent mass flow controllers (F-201CV-500 RGD-33-V, Bronkhorst High-Tech B.V., Gelderland, The Netherlands), resulting in defined partial pressures of each gas p_i,in_ at the inlet of the bioreactor.

Autotrophic batch cultivations were carried out by using a medium with 0.5 g L^−1^ cysteine-HCl as a reducing agent. The medium and the bioreactor were autoclaved (121 °C, 20 min) prior to inoculation and anaerobized for at least 12 h with the gas mixture under study. Shortly before inoculation, sterile L-cysteine-HCl stock solution was added aseptically via a septum (diameter 12 mm, Infors AG, Bottmingen, Switzerland) fixed in the lid of the bioreactor by use of a single-use syringe (BD Discardit II, Becton Dickinson, Franklin Lakes, NJ, USA) with microfiltration membrane (pore size 0.2 µm, VWR, Radnor, PA, USA) and sterile needles (Sterican 0.9 × 70 mm, B. Braun, Melsungen, Germany) in order to ensure anaerobic conditions and a low redox potential in the stirred-tank bioreactor. Inoculation was performed by using single-use syringes (BD Discardit II, Becton Dickinson, Franklin Lakes, NJ, USA) and injection through a septum (diameter 12 mm, Infors AG, Bottmingen, Switzerland) fixed at the top of the bioreactor to achieve an initial cell dry weight concentration of c_x,0_ = 0.05 g L^−1^ in studies with the mono-cultures. The same initial cell dry weight concentrations were applied in co-culture studies for each strain, resulting in a total initial cell dry weight concentration of c_x,0_ = 0.1 g L^−1^.

Studies with varying inlet CO partial pressures p_CO,in_ (0, 50, 100, and 800 mbar, respectively) were realized by varying the partial pressure of CO and adding N_2_ as make-up gas to keep the partial pressure of CO_2_ constant at p_CO2,in_ = 200 mbar.

In the process with the addition of sulfide, a feed rate of 0.05 mmol S^2−^ L^−1^ h^−1^ with a stock solution of 20 g L^−1^ Na_2_S·9 H_2_0 was adjusted using a peristaltic pump (Ismatec, Cole-Parmer GmbH, Wertheim, Germany). Sulfide feeding was started 24 h after inoculation of the batch processes.

**Figure 1:**
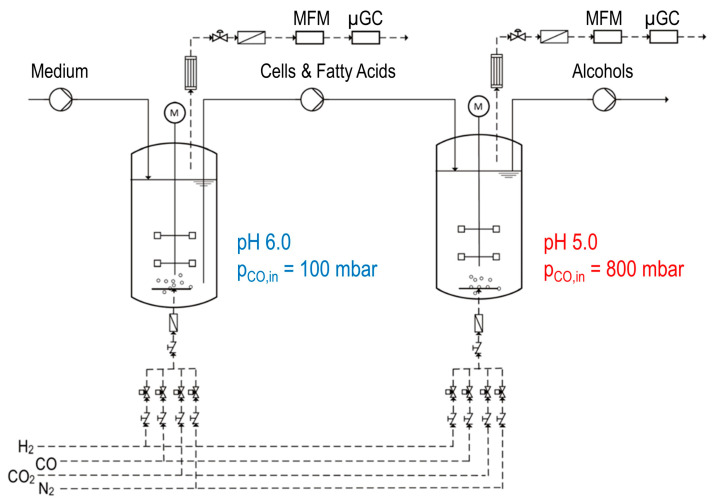
Scheme of the continuously operated stirred-tank reactors in series with continuous gas supply and exhaust gas analysis (mass flow meter (MFM), micro-gas chromatograph (µGC)) adapted with permission from Doll et al. [20]. 2018, Kathrin Doll.

For continuous cultivation of *C. carboxidivorans* and *C. kluyveri* in a cascade of two stirred-tank bioreactors, the STR with a total volume of 2.0 L was used as the first reactor with a working volume of 1.0 L and the STR with a total volume of 3.7 L was used as the second reactor with a working volume of 1.5 L. The first continuously operated stirred-tank reactor in the cascade was controlled to pH 6.0. The CO inlet partial pressure was set to 100 mbar at a gas composition of CO:CO_2_:N_2_ = 1:2:7, and the gas volume flow rate was set to 0.042 vvm. Additionally, the first reactor was fed with 0.05 mmol S^2−^ L^−1^ h^−1^ with a stock solution of 20 g L^−1^ Na_2_S ∙ 9 H_2_0 using a peristaltic pump (Ismatec, Cole-Parmer GmbH, Wertheim, Germany). The second reactor was controlled to pH 5.0. The CO inlet partial pressure was increased to 800 mbar at a gas volume flow rate of 0.083 vvm. The process temperature was set to 37 °C in both reactors. The dilution rates D of the continuously operated stirred-tank bioreactors were fixed to D_R1_ = 0.09 h^−1^ in the first reactor and D_R2_ = 0.06 h^−1^ in the second reactor. Continuous operation of the cascade was started after a 24-h batch phase after inoculation of the first reactor with a CDW concentration of 0.05 g L^−1^ of *C. carboxidivorans*, as well as *C. kluyveri* (in total 1.0 g L^−1^ dry cell mass). After initiating continuous operation of the cascade, inoculation of the second reactor took place. The configuration scheme of the stirred-tank cascade is pictured in Figure 1 adapted from Doll et al. [20].

### 2.4. Analytical Methods

For determination of biomass and product concentrations, 5 mL samples were frequently withdrawn aseptically by sterile single-use syringes (BD Discardit II, Becton Dickinson, Franklin Lakes, NJ, USA) applying sterile needles with a length of 12 cm (Sterican 0.8 × 120 mm, B. Braun, Melsungen, Germany) via a septum (diameter 12 mm, Infors AG, Bottmingen, Switzerland) fixed at the lid or the ports of the stirred-tank bioreactors. The optical density of the samples (OD_600_) was measured at 600 nm with a UV-Vis spectrophotometer (Genesys 10S UV-Vis, Thermo Scientific, Neuss, Germany) for the estimation of the cell dry weight (CDW) concentrations by using previously determined correlation factors [18]. Individual cell dry weight concentrations of *C. carboxidivorans* and *C. kluyveri* in co-culture were estimated based on the individual cell counts in the sample measured afterwards in solution fluorescence in situ hybridization (FISH) by flow cytometry (FC), as published before [18].

Product concentrations of organic acids and alcohols were measured by HPLC (Finnigan Surveyor, Thermo Fisher Scientific, Waltham, MA, USA) equipped with a refractive index (RI) detector (Finnigan Surveyor RI Plus Detector, Thermo Fisher Scientific, Waltham, MA, USA) and an Aminex-HPX-87H ion exchange column (Biorad, Munich, Germany). Separation of organic acids and alcohols was carried out at a constant flow rate of 0.6 mL min^−1^ of 5 mM H_2_SO_4_ as eluent at a column temperature of 60 °C (Figure S1). Prior to injection, HPLC samples were filtered with a 0.2 μm cellulose filter (Chromafil RC20/15 MS, Macherey-Nagel GmbH & Co., KG, Düren, Germany).

For the measurement of 1-hexanol, octanoate, and 1-octanol concentrations, samples were extracted by adding hexane (sample:hexane = 4:1), mixing for 15 min at 25 s^−1^ (Retsch MM200, RETSCH GmbH, Haan, Germany), and subsequent phase separation by centrifugation at 15,000 rcf for 3 min (Mikro 20, Hettich, Tuttlingen, Germany). An amount of 100 µL of the organic phase was then removed, mixed with 50 µL of an internal standard (1 g L^−1^ 1-pentanol in hexane) and used for GC analysis. At the end of the extraction, the samples were sealed gas-tight to avoid evaporation of hexane. The gas chromatographic (GC) analysis was automated using an autosampler (AOC-20s, Shimadzu Corp., Kyoto, Japan) and a GC auto-injector (AOC-20i, Shimadzu Corp., Kyoto, Japan), which took a sample volume of 5 µL with a split of the injector of 50. The stationary phase was a GC capillary column (FameWax, Restek GmbH, Bad Homburg, Germany), and the mobile phase (4.35 mL min^−1^; 4.96 bar) consisted of helium. The column temperature was increased from 50 °C to 180 °C at 7 °C min^−1^ after each sample injection. After the products were separated on the GC capillary column, they were detected by a flame ionization detector (FID-2010 Plus, Shimadzu Corp., Kyoto, Japan) operated at a temperature of 250 °C, with flow rates of 40 mL min^−1^ (H_2_) and 400 mL min^−1^ (He), respectively (Figure S2).

Mass flow meters were used for online measurement of the volumetric waste gas flow rate of both bioreactors (F-111B-1K0-RGD-33-E and F-101D-RAD-33-V, Bronkhorst High-Tech B.V, Ruurlo, The Netherlands). The composition of the exhaust gas could be analyzed online by an automated µ-gas chromatograph (µ 490, Agilent Technologies, Waldbronn, Germany). The µ-GC had three channels, which made it possible to detect different gaseous components at the same time. On the first column (molecular sieve column with carrier gas argon), gases such as He, H_2_, N_2_, O_2_, CO and short-chain alkanes could be detected. The second column (PPQ column with carrier gas helium) was used to detect CO_2_, NH_3,_ and NOx. The third channel (CP-Sil 5 column with carrier gas helium) allowed the gases H_2_S, C_2_S, and COS to be measured. A reflux condenser at the top of the stirred-tank reactors operated at 2 °C was used to minimize evaporation of water and alcohols from the bioreactors.

## 3. Results and Discussion

### 3.1. CO-Inhibition Studies with C. kluyveri in Stirred-Tank Reactors

**Figure 2:**
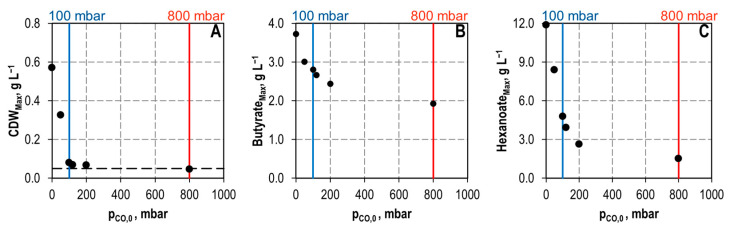
Maximum CDW concentrations (**A**), maximum butyrate concentrations (**B**) and maximum hexanoate concentrations (**C**) as function of varying CO partial pressures during batch cultivations of *C. kluyveri* with 15 g L^−1^ ethanol and 6 g L^−1^ acetate in a fully controlled stirred-tank bioreactor. Black dotted line shows the initial CDW concentration (T = 37 °C; pH = 6.0; P V^−1^ = 11.7 W L^−1^; F_gas_ = 0.083 vvm with 200 mbar CO_2_).

The CO sensitivity of *C. kluyveri* was studied with a mono-culture in batch-operated stirred-tank reactors with an initial supply of 6 g L^−1^ acetate and 15 g L^−1^ ethanol, respectively, at varying partial pressures (p_CO_) of up to 800 mbar CO in the gas phase. The resulting maximum CDW concentrations as well as the maximum butyrate and hexanoate concentrations are shown in Figure 2 as a function of the adjusted CO-partial pressures in the individual batch processes. Already a p_CO_ of 100 mbar reduced the maximum CDW concentration of *C. kluyveri* by 86% compared to a batch process without CO addition. At 800 mbar CO total growth inhibition of *C. kluyveri* was observed, but the CDW concentration of *C. kluyveri* was stable for 144 h. Maximum hexanoate concentrations were reduced by 60% at 100 mbar CO, and by 87% at 800 mbar CO, respectively, compared to a batch process without CO in the gas phase. Reduction of the maximum butyrate concentrations with increasing p_CO_ was less compared to the hexanoate concentrations (24% at 100 mbar CO, and 48% at 800 mbar CO, respectively).

These findings explain the behaviour of *C. kluyveri* during autotrophic co-cultivations with *C. carboxidivorans* at varying CO partial pressures in the inlet gas [17]. At 800 mbar CO_in_, chain elongation activity was observed, although growth of *C. kluyveri* was negligible. Organic acids produced by *C. kluyveri* were reduced by *C. carboxidivorans* to the corresponding alcohols. At 100 mbar CO_in_, growth of *C. kluyveri* was observed; however, the capacity of *C. carboxidivorans* to form alcohols was reduced, and a constant decay of *C. carboxidivorans* was observed [17].

**Figure 3:**
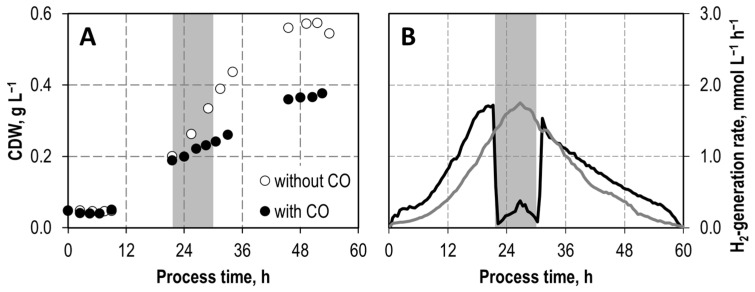
Batch cultivations of *C. kluyveri* in fully controlled stirred-tank bioreactors without CO in the inlet gas (open circles, grey line) and with gassing of 100 mbar CO indicated by the grey shaded area over a period of 9 h (black). (**A**) Cell dry weight concentrations (CDW); (**B**) Hydrogen-generation rate (T = 37 °C; pH = 6.0; P V^−1^ = 10.5 W L^−1^; F_gas_ = 0.042 vvm with 200 mbar CO_2_).

The inhibitory effect of CO on *C. kluyveri* was additionally investigated by performing a batch process with *C. kluyveri* in a continuously gassed stirred-tank reactor at pH 6.0 with a time-limited CO supply. Here, gassing with 100 mbar CO was started during exponential growth after a process time of 22 h. Nine hours later, the CO supply was switched off (Figure 3). The process data are compared with a reference experiment in which CO was not added at any time.

Up to the start of CO gassing, the CDW concentrations and the hydrogen generation rates were comparable in both processes. After initiation of the CO gassing, however, the growth rate decreased, and the hydrogen generation rate dropped from 1.6 mmol L^−1^ h^−1^ to 0.1 mmol L^−1^ h^−1^. Immediately after switching off the CO supply, the hydrogen generation rate was fully restored. The linear increase of the CDW concentrations was not affected by switching off the CO supply. As a consequence, a CDW concentration of only 70% of the reference process was observed after a process time of 53 h.

According to Seedorf et al. (2008) [14], the ferredoxin-dependent hydrogenase of *C. kluyveri* has a very high sequence similarity to the ferredoxin-dependent hydrogenase of *Clostridium pasteurianum*. For the latter, inhibition by exogenously added carbon monoxide was observed in studies with cell extracts of *C. pasteurianum* [24]. The ferredoxin-dependent hydrogenase in *C. kluyveri* is responsible for the production of hydrogen, whereby reduced ferredoxin is oxidized, thus regenerating the oxidized form of the cofactor [14]. Inhibition of the hydrogenase of *C. kluyveri* was also shown in the batch experiment as H_2_ formation immediately stopped after initiation of the gassing with 100 mbar CO. Since hydrogen production returned to the same level when CO gassing ceased, a reversible inhibition of the ferredoxin-dependent hydrogenase in *C. kluyveri* may be assumed.

As hydrogen formation can serve as an indicator of the metabolic activity of *C. kluyveri* [18], growth was also expectedly reduced during gassing with CO. This is the consequence of a stalling of energy metabolism because redox equivalents from reduced ferredoxin accumulate, which leads to a reduced growth rate. Interestingly, although upon CO removal hydrogen production increased again to the same level as before, the same CDW concentration could not be reached after 53 h, compared to the reference batch process. However, the results of the inhibition studies with continuous gassing (Figure 2) showed that growth is not essential for successful chain elongation by *C. kluyveri*, since even at 800 mbar CO the cell concentration was not reduced within a process time of 144 h, and butyrate as well as hexanoate were produced by the resting cells.

### 3.2. Continuous Sulfide Supply in Autotrophic Batch Processes with C. carboxidivorans

Since maintaining low CO partial pressures necessary for growth of *C. kluyveri* results in a reduced capacity of *C. carboxidivorans* to form alcohols and even the decay of *C. carboxidivorans* in co-culture occured [17], but on the other hand a 2:1 mixture of ethanol and acetate is favorable for chain elongation by *C. kluyveri*, a possibility to improve ethanol production was explored. To this end, the effects of a continuous sulfide supply were studied in batch-operated stirred-tank reactors with continuous gassing with *C. carboxidivorans.* An additional supply of sulfide was reported to increase alcohol formation in autotrophic processes, e.g., with *C. ragsdalei* [21].

**Figure 4:**
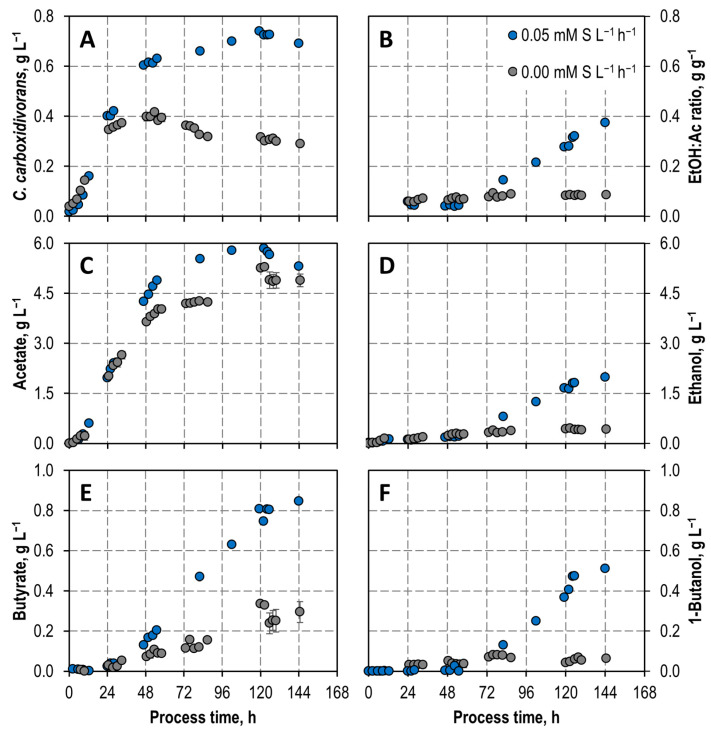
Autotrophic batch cultivations of *C. carboxidivorans* in continuously gassed, fully controlled stirred-tank bioreactors (T = 37 °C; pH = 6.0; P V^−1^ = 10.5 W L^−1^; 200 mbar CO_2_) with an initial CO partial pressure p_CO,in_ = 100 mbar at F_gas_ = 0.042 vvm without continuous sulfide supply (grey) compared to the same autotrophic batch process with continuous sulfide supply of 0.05 mM S L^−1^ h^−1^ (blue). (**A**) Cell dry weight concentrations (CDW) of *C. carboxidivorans*; (**B**) ethanol: acetate ratio; (**C**) acetate concentrations; (**D**) ethanol concentrations; (**E**) butyrate concentrations; (**F**) butanol concentrations.

Autotrophic batch processes were performed with *C. carboxidivorans* with and without continuous sulfide feeding of 0.05 mM S L^−1^ h^−1^ at a reduced CO partial pressure of p_CO,in_ = 100 mbar, applying continuously gassed stirred-tank bioreactors (Figure 4).

The growth of *C. carboxidivorans* started immediately after inoculation and reached a maximum CDW concentration of 0.72 g L^−1^ after 120 h showing a 2.2-fold increase, compared to the process without an additional sulfide supply. Initial acetate formation was the same compared to the process without sulfide feeding. After 33 h, more acetate was formed with sulfide supply. Initial ethanol formation was comparable to the process without sulfide feeding within the first 55 h. Afterwards, ethanol production was very much improved due to the additional sulfide supply, resulting in 4.7 times higher ethanol concentration after 144 h. The final ethanol-to-acetate ratio was thus improved by a factor of 4.1 (0.37 g ethanol per gram acetate which corresponds to 0.49 mol ethanol per mol acetate). Butyrate and 1-butanol production were improved by sulfide feeding as well. After 144 h a final concentration of 0.85 g L^−1^ butyrate, and 0.5 g L^−1^ 1-butanol, respectively, were measured (increase by a factor of 2.8 and 8, respectively, compared to the process without sulfide feeding).

**Figure 5:**
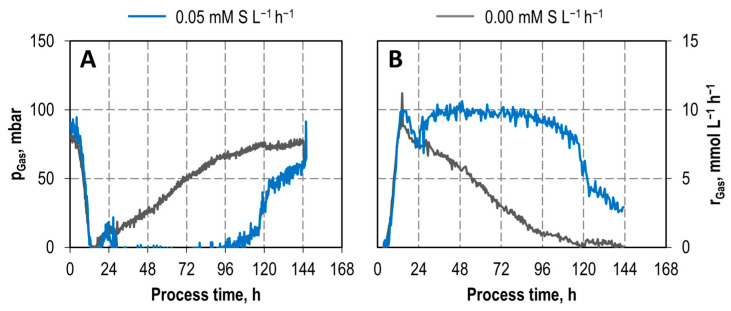
Partial pressures (**A**) and CO consumption rates (**B**) in stirred-tank reactors with *C. carboxidivorans* with sulfide feeding of 0.05 mM S L^−1^ h^−1^ (blue) and without sulfide feeding (grey) (T = 37 °C; pH = 6.0; P V^−1^ = 10.5 W L^−1^; F_gas_ = 0.042 vvm (10:20:70 CO:CO_2_:N_2_)).

Comparing CO conversion of the autotrophic batch processes of *C. carboxidivorans* with and without continuous sulfide feeding at reduced CO partial pressure of 100 mbar (Figure 5) shows nearly complete CO conversion between 12–120 h with sulfide feeding, whereas nearly complete CO conversion was only observed between 12 and 24 h without sulfide feeding. Since the continuous supply of sulfide was started after a process time of 24 h, the partial pressures as well as the CO uptake rate were comparable within the first 24 h. After that, the CO uptake rate showed a continuously high uptake of 10 mmol L^−1^ h^−1^ with a continuous sulfur supply, whereas a continuous reduction of the CO uptake rate was recorded without a sulfur supply. The uptake of CO was limited due to the low partial pressure and the subsequent complete uptake of CO. In total, 1107 mmol CO was consumed by *C. carboxidivorans* with an additional sulfide supply compared to 461 mmol CO without sulfide feeding, which corresponds to an increase by a factor of 2.4. Carbon balances were closed to 99% and 97%, respectively.

Even compared with an autotrophic batch process with *C. carboxidivorans* at 800 mbar initial CO with continuous gassing (0.083 vvm)—the conditions for improved formation of alcohols [17]—the final CDW concentration was increased by a factor of 1.7 at 100 mbar initial CO with sulfide feeding, and the maximum acetate concentration was improved by a factor of 3 (5.8 g L^−1^ after 120 h). The same final ethanol concentration was achieved at low initial CO partial pressure with continuous sulfide feeding compared to the autotrophic batch process with the high initial CO partial pressure of 800 mbar. Butyrate and 1-butanol concentrations were increased by sulfide feeding at a low CO partial pressure by factors of 6 and 2, respectively [18].

An increase in biomass formation in the presence of sulfide has previously been observed with the facultatively anaerobic bacterium *Rhodospirillum rubrum* due to stimulation of the CO dehydrogenase [25]. H_2_S finds particular application as a reducing agent, as it manages to lower the redox potential of the medium by electron donation. Here, it was observed that the metabolism of clostridia shifts towards solventogenesis. This is related to the availability of more reducing equivalents to form NADH for the conversion of acetyl-CoA to ethanol. The reducing agent thus causes a redirection of carbon flux to the formation of ethanol instead of acids [26,27]. The shift of metabolism towards solventogenesis has already been observed with *C. ljungdahlii* [27].

Compared with an autotrophic batch process with *C. ragsdalei* with the same sulfide feeding rate [21], the increase in the formation of ethanol was clearly higher with *C. carboxidivorans*, compared to batch processes without sulfide feeding. A nearly five times higher ethanol concentration was measured after 144 h compared to the more than three-fold increase in the final ethanol concentration with *C. ragsdalei.* But the striking difference between these two acetogens was the ethanol-to-acetate ratio achieved with sulfide feeding. With *C. ragsdalei* a final ethanol-to-acetate ratio of 17.7 g g^−1^ was observed, whereas the autotrophic batch process with *C. carboxidivorans* resulted in a ratio of only 0.37 g g^−1^.

It is known that a higher alcohol-to-acid ratio leads to a better chain elongation by *C. kluyveri* and thus to an increased formation of hexanoate compared to butyrate [12]. The continuous addition of sulfide resulted in an improved molar ethanol-to-acetate ratio of 0.4 compared to 0.09 without sulfur. Nevertheless, the achieved improvement in the ethanol-to-acetate ratio with sulfide feeding does not correspond to a desired ratio of at least 2 mol ethanol per mol acetate. An increased ethanol-to-acetate ratio leads to increased CDW concentrations of *C. kluyveri*, due to increased ATP production by ethanol oxidation [28,29]. When the ratio is reduced, chain elongation is thought to become more energy efficient at the expense of growth, compensating for reduced ATP production [11,30]. To the best of our knowledge, no previous study regarding the ideal substrate ratio of *C. kluyveri* has included the effects of CO on the metabolism of *C. kluyveri*. Previous studies as well as the data shown here on CO inhibition of *C. kluyveri* have demonstrated that chain elongation to hexanoate is quite possible despite restricted growth [18]. Therefore, it is assumed that, from the point of view of additional CO gassing, an “ideal” ethanol-to-acetate ratio is less relevant than a sufficiently high concentration of ethanol to meet the energy demand of the cells by chain elongation. With the continuous supply of sulfide, the formation of ethanol by *C. carboxidivorans* could be strongly increased, so that an improved initial situation for chain elongation by *C. kluyveri* is provided.

### 3.3. Co-Cultivation Studies in Two Bioreactors in Series with Varying pH and pCO

As growth of *C. kluyveri* is inhibited by increased CO partial pressures, but ethanol formation of *C. carboxidivorans* is reduced at low CO partial pressures, the first continuously operated stirred-tank bioreactor of the cascade was operated with an initial pCO of 100 mbar with an additional sulfide supply of 0.05 mmol S^2−^ L^−1^ h^−1^ to enhance ethanol formation of *C. carboxidivorans.* The first reactor was operated at pH 6.0 to enable growth of both organisms and chain elongation activity of *C. kluyveri*. The pH in the second reactor of the cascade was reduced to pH 5 to ensure increased alcohol formation by *C. carboxidivorans*, since this ability is coupled to low pH [20]. The partial pressure of CO in the gas phase was increased to 800 mbar in the second stirred-tank bioreactor to further boost the reduction of the organic acids to the corresponding alcohols by *C. carboxidivorans.*

**Figure 6:**
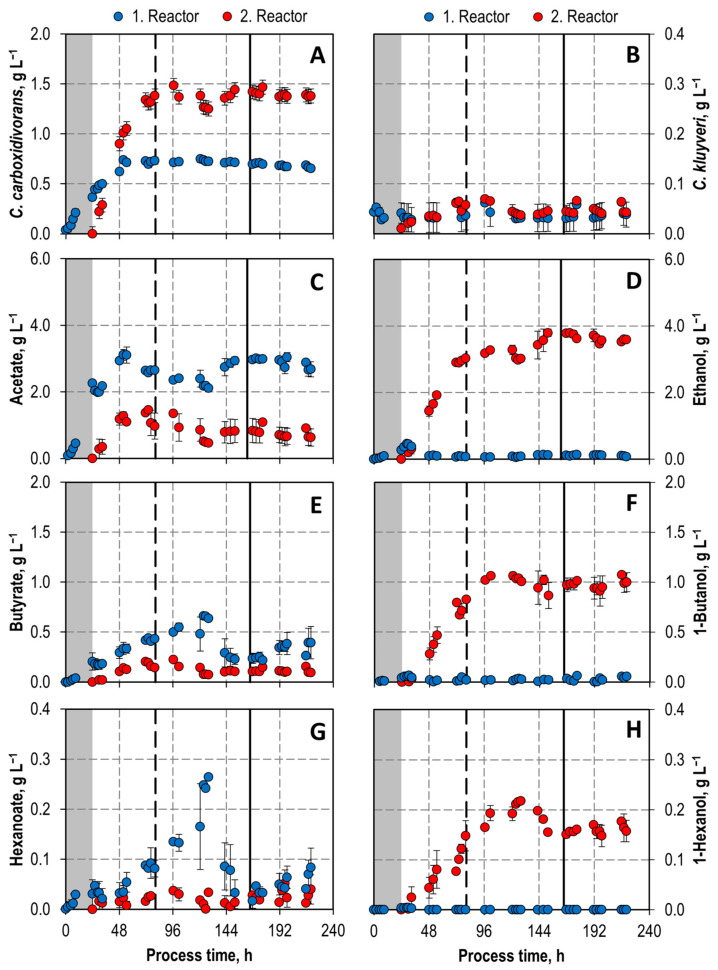
Biomass ((**A**): *C. carboxidivorans*; (**B**): *C. kluyveri*) and product concentrations ((**C**): acetate; (**D**): ethanol; (**E**): butyrate; (**F**): 1-butanol; (**G**): hexanoate; (**H**): 1-hexanol) during autotrophic co-cultivation of *C. carboxidivorans* and *C. kluyveri* in a continuously operated stirred-tank bioreactor cascade with a dilution rate of 0.09 h^−1^ in the first reactor and 0.06 h^−1^ in the second reactor (P = 1 bar, T = 37 °C, pCO_2,in_ = 200 mbar CO_2_). First reactor (blue symbols): pH 6.0, p_CO,in_ = 100 mbar, F_gas_ = 0.042 vvm, 0.05 mM S L^−1^ h^−1^; second reactor (red symbols): pH 5.0, p_CO,in_ = 800 mbar, F_gas_ = 0.083 vvm. Symbols show the mean and the minimum and maximum data of two independent gas fermentation experiments performed in the bioreactor cascade. The initial batch phase is shaded in grey. Vertical lines indicate process time of five mean hydraulic residence times of the first reactor (dashed line) and of the bioreactor cascade (solid line).

**Figure 7:**
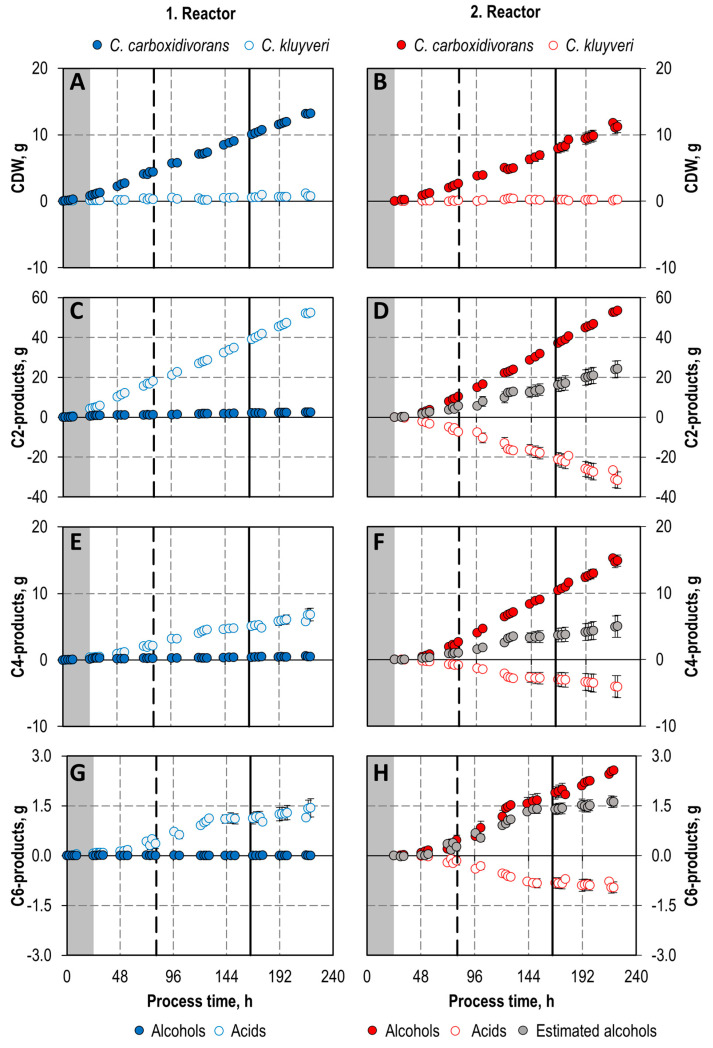
Cumulative production/consumption of cells ((**A**): CDW of the 1. Reactor; (**B**): CDW of the second reactor) and products ((**C**,**E**,**G**): C2,C4 and C6 products of the first reactor; (**D**,**F**,**H**): C2,C4 and C6 products of the second reactor) during autotrophic co-cultivation of *C. carboxidivorans* and *C. kluyveri* in a continuously operated stirred-tank bioreactor cascade with a dilution rate of 0.09 h^−1^ in the first reactor and 0.06 h^−1^ in the second reactor (P = 1 bar, T = 37 °C, pCO_2,in_ = 200 mbar CO_2_). First reactor (blue): pH 6.0, p_CO,in_ = 100 mbar, F_gas_ = 0.042 vvm, 0.05 mM S L^−1^ h^−1^; second reactor (red): pH 5.0, p_CO,in_ = 800 mbar, F_gas_ = 0.083 vvm. Alcohols (ethanol, 1-butanol, 1-hexanol) as well as CDW of *C. carboxidivorans* are shown in full circles; acids (acetate, butyrate, hexanoate) as well as CDW of *C. kluyveri* are shown in open circles. Grey symbols show the estimated production of alcohols if the corresponding organic acids consumed in the second reactor will be reduced solely. The difference between these estimated cumulative productions of alcohols and the measured ones show de novo synthesis. Symbols show the mean and the minimum and maximum data of two independent gas fermentation experiments performed in the bioreactor cascade. The initial batch phase is shaded in grey. Vertical lines indicate process time of five mean hydraulic residence times of the first reactor (dashed line) and of the bioreactor cascade (solid line).

The CDW concentrations of *C. carboxidivorans* (Figure 6), measured in the co-culture via *in solution* fluorescence in situ hybridization (FISH) by flow cytometry (FC), increased rapidly in both the first and the second reactor after switching to continuous operation. After 2 days, a constant concentration of *C. carboxidivorans* (0.68 ± 0.01 g L^−1^) was observed in the first reactor. Two days later, after 4 days, the concentration of *C. carboxidivorans* became nearly constant in the second stirred-tank bioreactor with a doubled CDW concentration of 1.42 ± 0.04 L^−1^ in the steady state after five hydraulic residences times of the cascade. Thus, biomass formation of *C. carboxidivorans* in the co-culture was nearly the same in both bioreactors despite the differing reaction conditions. However, the cumulative net production of *C. carboxidivorans* cells in the two bioreactors shows a reduced biomass production rate in the second reactor due to the reduced dilution rate (Figure 7).

Growth of *C. kluyveri* was observed in the first continuously operated bioreactor with a mean growth rate of 0.09 h^−1^ (growth rate = dilution rate in the steady state), as the biomass concentration of *C. kluyveri* was kept more or less stable throughout the whole process time of 196 h continuous operation, whereas growth of *C. kluyveri* was negligible in the second stirred-tank bioreactor (Figure 6), but the *C. kluyveri* cells were stable under these unfavorable reaction conditions (low pH, high CO concentrations) (Figure 7).

Acetate, butyrate and hexanoate formation was observed in the first bioreactor (Figure 7), reaching more or less stable concentrations of 2.89 ± 0.09 g L^−1^ acetate, 0.31 ± 0.07 g L^−1^ butyrate, and 0.05 ± 0.01 g L^−1^ hexanoate, respectively, after 12.5 mean hydraulic residence times (Figure 6). Mean formation rates are summarized in Table 1.

In contrast, no formation of the alcohols ethanol, 1-butanol, and 1-hexanol was observed in the first stirred-tank bioreactor of the continuous co-culturing process, whereas alcohol formation took place in the second reactor solely (Figure 7, Table 1). Final steady state concentration of 3.78 g L^−1^ ethanol, 0.97 ± 0.07 g L^−1^ 1-butanol, 0.16 ± 0.01 g L^−1^ 1-hexanol, and 0.77 ± 0.03 mg L^−1^ 1-octanol were measured in the product solution of the cascade (Figure 6). The organic acids produced in the first bioreactor were not fully reduced to the corresponding alcohols in the second bioreactor of the cascade, but high alcohol/acid ratios were achieved (4.5 g ethanol g^−1^ acetate, 9.1 g 1-butanol g^−1^ butyrate, and 6.8 g 1-hexanol g^−1^ hexanoate, respectively).

In addition to the reduction of the organic acids produced in the first reactor, de novo synthesis of ethanol, 1-butanol, and 1-hexanol, respectively, was observed in the second stirred-tank bioreactor (Figure 7). Ethanol amounting to 121% more, 195% more 1-butanol, and 58% more 1-hexanol were produced after a process time of 220 h.

Thus, a clear spatial separation of acidogenesis in the first bioreactor and solventogenesis in the second bioreactor was achieved with this continuous cascading co-cultivation process with *C. carboxidivorans* and *C. kluyveri* (Figure 7). This was only possible because *C. kluyveri* consumed all the ethanol produced by *C. carboxidivorans* for chain elongation in the first stirred-tank bioreactor. Previously published CO fermentation data with a monoculture of *C. carboxidivorans* in a continuously operated stirred-tank bioreactor at a dilution rate of 0.12 h^−1^ at pCO, in = 800 mbar showed that *C. carboxidivorans* produces ethanol and acetate at a 1:1 ratio already in the acidogenic phase [20]. Chain elongation by *C. kluyveri* in the co-culture resulted in much higher butyrate concentrations (0.31 g L^−1^ compared to 0.08 g L^−1^) in the steady state and even enabled considerable hexanoate formation compared to the published data with a monoculture of *C. carboxidivorans* [20].

In addition, a hydrogen gas production rate of 1.2 mmol L^−1^ h^−1^ was measured in the first reactor of the continuous co-cultivation process beginning at a process time of 32 h. This is typical for chain-elongating *C. kluyveri*. As already shown with a monoculture of *C. carboxidivorans* at the low p_COin_ of 100 mbar with sulfide feeding in a batch process (Figure 5), CO was completely consumed by *C. carboxidivorans* in the continuous co-cultivation process beginning at a process time of 30 h in the first reactor, thus reducing CO inhibition of *C. kluyveri*.

In contrast to the very much reduced CO inhibition of *C. kluyveri* in the first reactor of the continuous co-cultivation process, biomass formation rate was exceptionally low (Figure 7). This is most probably due to a severe substrate limitation of *C. kluyveri* in the co-cultivation process, because ethanol production by *C. carboxidivorans* is low under the reaction conditions of the first bioreactor of the cascade as shown before with a final ratio of 0.37 g ethanol g^−1^ acetate produced in a batch process (Figure 4). Even the high biomass ratio of *C. carboxidivorans* to *C. kluyveri* of 17:1 in the steady state in the first bioreactor of the cascade was not sufficient to provide enough of the limiting substrate ethanol to improve biomass formation by the chain-elongating *C. kluyveri* in co-culture.

Compared to a batch process with the identical co-culture at a high initial CO partial pressure of 800 mbar, the final steady state concentrations of ethanol and 1-butanol were increased by factors of 1.8 and 1.2, respectively, in the steady state (Table 1). This is a surprising result because usual product concentrations are higher in batch compared to continuous bioprocesses. In contrast, the steady state concentrations of the organic acids were clearly reduced in the cascade compared to the batch process due to the optimum conditions for the reductive formation of alcohols by *C. carboxidivorans* (pH 5.0, 800 mbar CO).

As a consequence, the space-time yields of the alcohols were very much improved with the continuously operated stirred-tank bioreactor cascade compared to the batch process. Volumetric ethanol, 1-butanol, and 1-hexanol production rates were increased by a factor of 5.3, 2.7, and 1.9, respectively (Table 1).

Another co-culture with the acetogenic bacterium *Clostridium ljungdahlii* with the chain-elongating bacterium *C. kluyveri* in a continuously operated stirred-tank bioreactor with a continuous syngas supply (60% CO, 35% H_2_ and 5% CO_2_) at a bleed rate of 25% of the applied dilution rate resulted in a hexanoate production rate of 1.3 g L^−1^ d^−1^ at pH 6.0, which corresponds to an increase by the factor 11.8 compared to our results. But the hexanoate was reduced to 1-hexanol with only a two-fold increased volumetric productivity of 0.3 g L^−1^ d^−1^ by *C. ljungdahlii* after ~1300 h, compared to the hexanol productivity of the cascade after 167 h of process time [31].

**Figure 8:**
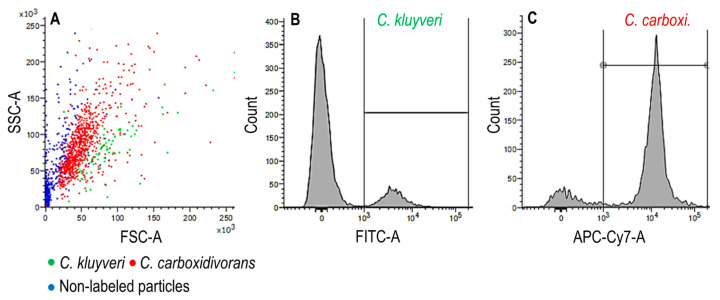
FISH-FC sample after 167 h from the first reactor of a continuously operated stirred-tank bioreactor cascade with *C. carboxidivorans* and *C. kluyver*. (**A**) Scattergram with the intensity of both fluorescence signals (*C. kluyveri* in green and *C. carboxidivorans* in red). Blue dots indicated other, non-labeled particles. The axes show the intensity of the fluorescence signal (arbitrary units). (**B**) Histograms with the cell counts of *C. kluyveri* vs. intensity of the FITC-fluorescence signal excited by a blue laser at 488 nm and detected using the 527/32 nm band-pass filter (FITC) gated between a fluorescence intensity of 10^3^–10^5^. (**C**) Histograms with the cell counts of *C. carboxidivorans* vs. intensity of the Cy5-fluorescence signal excited by red laser at 640 nm and detected by using the 660/10 nm band-pass filter (APC-Cy7) gated between a fluorescence intensity of 10^3^–10^5^ (A = Area).

**Figure 9:**
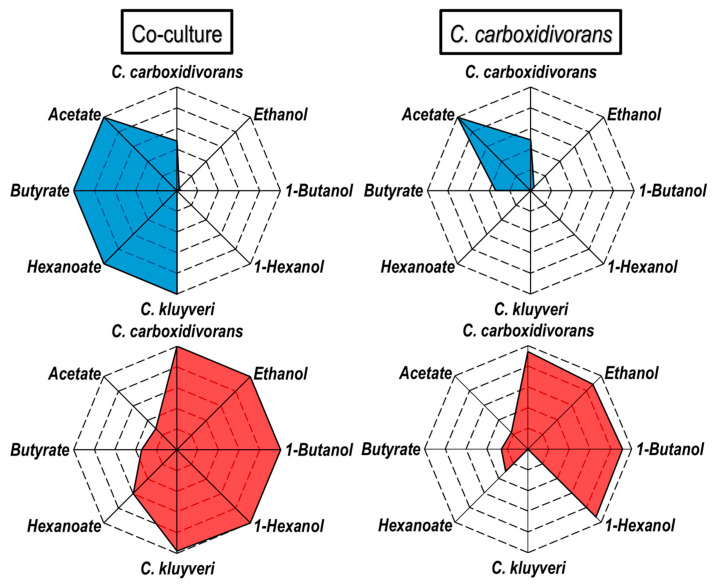
Comparison of steady state concentrations of continuous autotrophic CO-conversion processes applying a stirred-tank bioreactor cascade with a dilution rate of 0.09 h^−1^ in the first reactor and 0.06 h^−1^ in the second reactor (P = 1 bar, T = 37 °C, pCO_2,in_ = 200 mbar CO_2_). (**Left**): co-cultivation of *C. carboxidivorans* and *C. kluyveri.* (**Right**): monoculture of *C. carboxidivorans.* First reactor (blue): pH 6.0, p_CO,in_ = 100 mbar, F_gas_ = 0.042 vvm, 0.05 mM S L^−1^ h^−1^; second reactor (red): pH 5.0, p_CO,in_ = 800 mbar, F_gas_ = 0.083 vvm. The highest concentration achieved regardless of the first or the second reactor was normalized to 1.

As slow growth of *C. kluyveri* was only observed in the first continuously operated stirred-tank bioreactor of the cascade and there was no growth of *C. kluyveri* in the second bioreactor, resulting in a high steady state biomass ratio of *C. carboxidivorans* to *C. kluyveri* of 17:1 (Figure 8) in the first and 35.5:1 in the second reactor, the process performance of a *C. carboxidivorans* monoculture was studied in the cascade as well as under identical reaction conditions to compare the results with the continuous co-culture. The final product concentrations measured in both processes in the steady states at a process time of 167 h are compared in Figure 9. The monoculture of *C. carboxidivorans* produced almost exclusively acetate in the first stirred-tank reactor due to the low CO partial pressure, whereas butyrate and hexanoate were produced in the co-culture process by *C. kluyveri* via chain elongation resulting in a three-times higher concentration of butyrate in the first reactor and enabling hexanoate production as well. Due to the increased fatty acid concentrations produced in the first bioreactor, about 10% higher alcohol concentrations were achieved in the second reactor with the continuous co-culture, compared to the monoculture of *C. carboxidivorans* (Figure 9). In addition, butyrate and hexanoate concentrations were higher in the second reactor of the co-cultivation process, demonstrating that the mean hydraulic residence time may not have been high enough to enable full conversion of the organic acids coming from the first stirred-tank bioreactor. Or conversely, the capability of *C. carboxidivorans* for the de novo synthesis of alcohols from CO was quite high under the reaction conditions chosen in the second bioreactor of the cascade.

## 4. Conclusions

Syngas fermentation with co-cultures of acetogenic and chain-elongating microorganisms are a promising approach for the conversion of CO to C2–C6 alcohols. Continuous production of these alcohols from CO was made possible applying a stable synthetic co-culture of *C. carboxidivorans* and *C. kluyveri* with two stirred-tank bioreactors in series. The separation of chain elongation and alcohol production by cascading of two continuously operated bioreactors resulted in the improved production of C2–C6-alcohols from CO compared to a monoculture of *C. carboxidivorans*. CO-inhibition of *C. kluyveri* was reduced at low CO-concentrations in the first bioreactor, and ethanol production of *C. carboxidivorans* was enhanced by additional sulfide supply at those unfavorable low CO concentrations. However, both measures were not sufficient to enable comparable biomass formation of both strains in co-culture. Steady state biomass concentrations of *C. carboxidivorans* were already 17 times higher in the first bioreactor of the continuous cascade compared to *C. kluyveri.* As a consequence, less CO-sensitive chain-elongating microorganisms are necessary in the future to improve continuous co-cultivation processes with acetogenic bacteria like *C. carboxidivorans* converting CO, since high CO partial pressures are necessary for the formation of beneficial ratios of ethanol to acetate for chain elongation by *C. kluyveri*, as well as for the reduction of carboxylic acids to the corresponding alcohols with *C. carboxidivorans*. In addition, high CO partial pressures are inevitable on an industrial scale at least at the lower part of the usually applied bubble-column reactors due to the hydrostatic pressure caused by liquid heights of up to 30 m.

## Figures and Tables

**Table 1:** Comparison of steady-state concentrations (c_ss_) and space-time yields (STY) of the continuously operated autotrophic stirred-tank reactor cascade with *C. carboxidivorans* and *C. kluyveri* after 167 h (Figure 5) and the batch co-culture of *C. carboxidivorans* and *C. kluyveri* [18]. Cascade: dilution rate of 0.09 h^−1^ in the first reactor and 0.06 h^−1^ in the second reactor (T = 37 °C, 200 mbar CO_2_). First reactor: pH 6.0, p_CO,in_ = 100 mbar, F_gas_ = 0.042 vvm, 0.05 mM S^2−^ L^−1^ h^−1^; second reactor: pH 5.0, p_CO,in_ = 800 mbar, F_gas_ = 0.083 vvm. Batch: p_CO,in_ = 800 mbar, F_gas_ = 0.083 vvm, pH 6.0, T = 37 °C. Carbon balances were closed with 99% carbon recovery.

	c_ss_, g L^−1^		c_final,_ g L^−1^	STY, g L^−1^ d^−1^			
	1. Reactor	2. Reactor	Batch [18]	1. Reactor	2. Reactor	Cascade	Batch [18]
*C. kluyveri*	0.04 ± 0.02	0.04 ± 0.01	0.06 ± 0.01	0.15 ± 0.05	0.03 ± 0.01	0.04 ± 0.02	0.03 ± 0.01
*C. carboxidivorans*	0.68 ± 0.01	1.42 ± 0.04	0.47 ± 0.03	1.78 ± 0.02	1.62 ± 0.16	1.24 ± 0.05	0.73 ± 0.02
Acetate	2.89 ± 0.09	0.84 ± 0.36	1.20 ± 0.02	6.62 ± 0.15	−4.56 ± 0.55	0.75 ± 0.31	4.15 ± 0.03
Ethanol	0.11 ± 0.01	3.78 ± 0.00	2.09 ± 0.20	0.28 ± 0.02	8.18 ± 0.14	3.12 ± 0.05	0.59 ± 0.00
Butyrate	0.31 ± 0.07	0.11 ± 0.05	0.31 ± 0.05	1.08 ± 0.14	−0.72 ± 0.25	0.11 ± 0.04	0.51 ± 0.03
1-Butanol	0.03 ± 0.01	0.97 ± 0.07	0.84 ± 0.06	0.04 ± 0.01	2.38 ± 0.12	0.89 ± 0.06	0.33 ± 0.03
Hexanoate	0.05 ± 0.01	0.03 ± 0.01	0.36 ± 0.03	0.33 ± 0.05	−0.24 ± 0.04	0.02 ± 0.01	0.12 ± 0.02
1-Hexanol	−	0.16 ± 0.01	0.24 ± 0.01	−	0.44 ± 0.01	0.15 ± 0.01	0.08 ± 0.01

## Data Availability

The original contributions presented in the study are included in the article/Supplementary Materials; further inquiries can be directed to the corresponding authors.

## Supplementary Materials

The following supporting information can be downloaded at: https://www.mdpi.com/article/10.3390/microorganisms11041003/s1, Table S1: Composition of the previously described medium [20] used for preculture and batch studies, Figure S1: Chromatograms of the organic acids and alcohols measured by HPLC (Finnigan Surveyor, Thermo Fisher Scientific, Waltham, USA) equipped with a refractive index (RI) detector (Finnigan Surveyor RI Plus Detector, Thermo Fisher Scientific, Waltham, USA) and an Aminex-HPX-87H ion exchange column (Biorad, Munich, Germany). Separation of organic acids and alcohols was carried out at a constant flow rate of 0.6 mL min^−1^ of 5 mM H_2_SO_4_ as eluent at a column temperature of 60 °C. (A) all products; (B) sample of the first reactor after 167 h; (C) sample of the second reactor after 167 h of process time.; Figure S2: Chromatograms of the gas chromatographic (GC) analysis, automated by using an autosampler (AOC-20s, Shimadzu Corp., Kyoto, Japan) and a GC auto-injector (AOC-20i, Shimadzu Corp., Kyoto, Japan), which took a sample volume of 5 µL with a split of the injector of 50. The stationary phase: GC capillary column (FameWax, Restek), mobile phase (4.35 mL min^−1^; 4.96 bar): helium. The column temperature was increased from 50 °C to 180 °C at 7 °C min^−1^ after each sample injection. After the products were separated on the GC capillary column, they were detected by a flame ionization detector (FID-2010 Plus, Shimadzu Corp., Kyoto, Japan) operated at a temperature of 250 °C with flow rates of 40 mL min^−1^ (H_2_) and 400 mL min^−1^ (He), respectively. (A) 1-Pentanol and 1-Hexanol; (B) 1-Pentanol and 1-Octanol; (C) 1-Pentanol and octanoate; (D) sample of the second reactor after 167 h with 1-Pentanol and 1-Hexanol. First peak is hexane.
