# Supplementary material for: Continuous Production of Ethanol, 1-Butanol and 1-Hexanol from CO with a Synthetic Co-Culture of Clostridia Applying a Cascade of Stirred-Tank Bioreactors
Source: Microorganisms. 2023 Apr 12;11(4):1003. doi: 10.3390/microorganisms11041003 (PMC10144111; doi:10.3390/microorganisms11041003)
Supplement: Supplementary file 1 [file microorganisms-11-01003-s001.zip › microorganisms-2313231-supplementary.pdf]

Supplementary material.

Table S1 Composition of the previously described medium [20] used for preculture and batch studies

**Component**	**Formula**	**Concentration in Stock solution, g L⁻¹**
**Mineral solution**		**33.3x**
Ammonium chloride	NH_4Cl	100
Sodium chloride	$NaCl$	80
potassium chloride	KCl	10
potassium dihydrogen phosphate	KH_2PO_4	10
Magnesium sulfate	$MgSO_4$	20
Calcium chloride	$CaCl_2$	4
**Vitamin solution**		**100x**
Pyridoxine	$C_8H_{11}NO_3$	0.01
Thiamine	$C_{12}H_{17}ClN_4OS$	0.005
Riboflavin	$C_{17}H_{20}N_4O_6$	0.005
Calcium pantothenate	$Ca(C_9H_{16}NO_5)_2$	0.005
Liponic acid	$C_8H_{14}O_2S_2$	0.005
Para amino benzoic acid	$C_7H_7NO_2$	0.005
Nicotinic acid	$C_6H_5NO_2$	0.005
Vitamin B12	$C_{72}H_{100}CoN_{18}O_{17}P$	0.005
D-biotin	$C_{10}H_{16}N_2O_3S$	0.002
Folic acid	$C_{19}H_{19}N_7O_6$	0.002
2-mercapto ethane sulfonic acid	$C_2H_6O_3S_2$	0.02
**Trace element solution**		**100x**

Nitrilotriacetic acid	$C_6H_9NO_6$	2.00
Manganese sulfate	$MnSO_4$	1.00
Ammonium iron sulfate	$NH_4Fe(SO_4)_2$	0.80
Cobalt chloride	$CoCl_2$	0.20
Zinc sulfate	$ZnSO_4$	0.20
Copper chloride	$CuCl_2$	0.02
Nickel chloride	$NiCl_2$	0.02
Sodium molybdate	Na_2MoO_4	0.02
Sodium selenate	Na_2SeO_4	0.02
Sodium wolframate	Na_2WO_4	0.02
		**Medium concentration**
**Yeast extract**		1.0 g L^{-1}
**Cysteine hydrochloride**	$C_3H_7NO_2S$ HCl	0.5 g L^{-1}
**Morpholino ethanesulfonic acid**[a)]	$C_6H_{13}NO_4S$	15.0 g L^{-1}
**Polypropylene glycol (1:10)**[b)]		0.1 mL L^{-1}

a) used only for heterotrophic preculture in anaerobic flasks

b) only for reactor studies

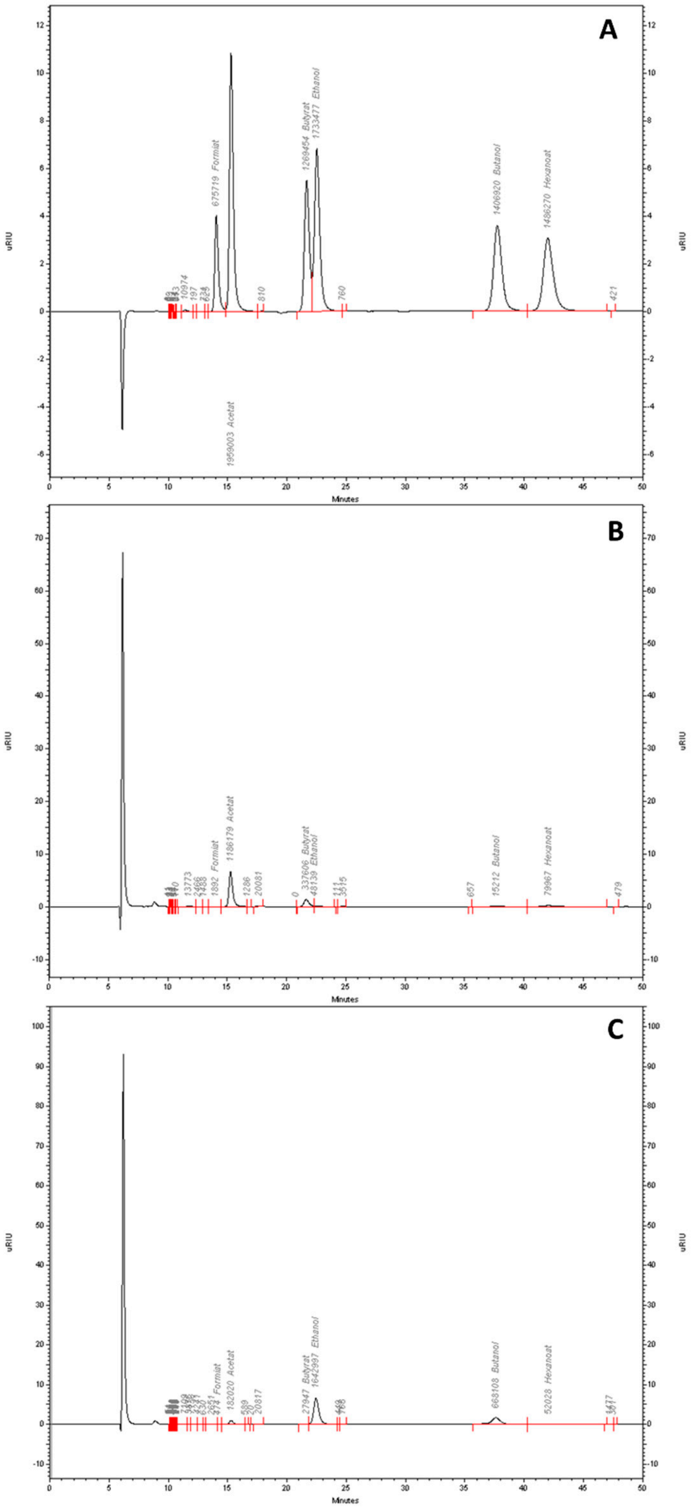

Figure S1 Chromatograms of the organic acids and alcohols measured by HPLC (Finnigan Surveyor, Thermo Fisher Scientific, Waltham, USA) equipped with a refractive in-dex (RI) detector (Finnigan Surveyor RI Plus Detector, Thermo Fisher Scientific, Waltham, USA) and an Aminex-HPX-87H ion exchange column (Biorad, Munich, Germany). Separation of organic acids and alcohols was carried out at a constant flow rate of 0.6 mL min^{-1} of 5 mM H_2SO_4 as eluent at a column temperature of 60°C. (A) all products; (B) sample of the first reactor after 167 h; (C) sample of the second reactor after 167 h of process time.

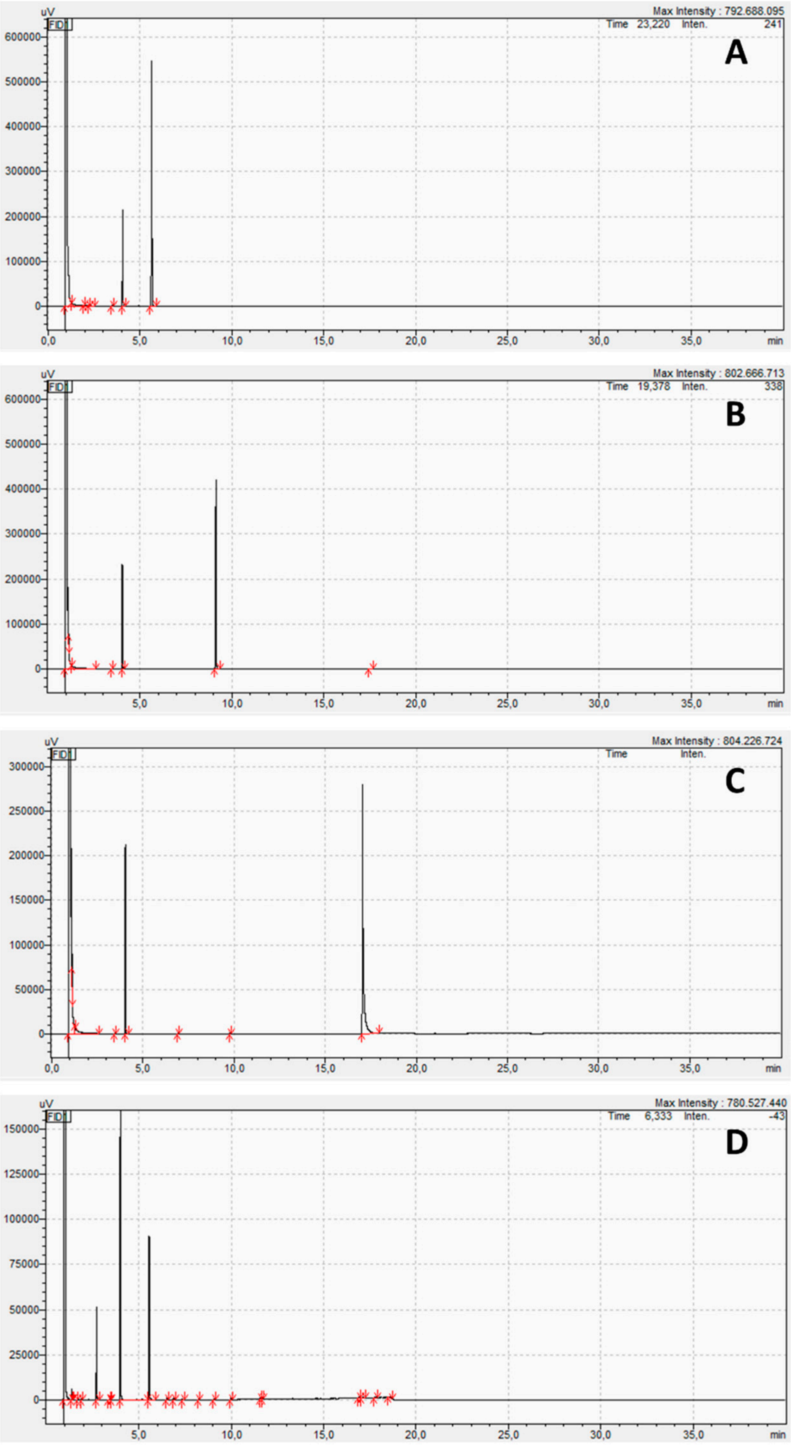

Figure S2 Chromatograms of the gas chromatographic (GC) analysis, automated by using an autosampler (AOC-20s, Shimadzu Corp., Kyoto, Japan) and a GC auto-injector (AOC-20i, Shimadzu Corp., Kyoto, Japan), which took a sample volume of 5 µL with a split of the injector of 50. The stationary phase: GC capillary column (FameWax, Restek), mobile phase (4.35 mL min^{-1}; 4.96 bar): helium. The column temperature was increased from 50 °C to 180 °C at 7 °C min^{-1} after each sample injection. After the products were separated on the GC capillary column, they were detected by a flame ionization detector (FID-2010 Plus, Shimadzu Corp., Kyoto, Japan) operated at a temperature of 250 °C with flow rates of 40 mL min^{-1} (H_2) and 400 mL min^{-1} (He), respectively. (A) 1-Pentanol and 1-Hexanol; (B) 1-Pentanol and 1-Octanol; (C) 1-Pentanol and octanoate; (D) sample of the second reactor after 167 h with 1-Pentanol and 1-Hexanol. First peak is hexane.
